# Treatment of septic shock in two pediatric patients with severe diabetic ketoacidosis using invasive hemodynamic monitoring: a case report

**DOI:** 10.1186/s12902-023-01315-4

**Published:** 2023-03-20

**Authors:** Amir Saeed, Fateme Ziyaee

**Affiliations:** 1 Division of Intensive Care Unit, Department of Pediatrics, Shiraz University of Medical Sciences, Shiraz, Iran; 2 Department of Pediatrics, Shiraz University of Medical Sciences, Zand Ave., Shiraz, Iran

**Keywords:** Diabetic ketoacidosis, Invasive hemodynamic monitoring, Septic shock, Case report

## Abstract

**Background:**

Diabetic ketoacidosis (DKA) is a life-threatening complication of diabetes mellitus. DKA associated with shock is a rare condition that occurs due to the fluid deficit or septic shock. It is not easy to differentiate these two conditions by clinical judgment and laboratory findings. Although the fluid therapy is the mainstay in DKA treatment, it looks like a double-edged sword—underhydration may result in organ failure whereas overhydration may lead to pulmonary and cerebral edema (CE).

**Case presentation:**

Herein, we report on two pediatric patients presenting with DKA and septic shock. The first patient was an 8-year-old boy newly diagnosed with type 1 diabetes mellitus (T1DM) who presented with DKA and septic shock. We used a device for continuous hemodynamic monitoring (proAQT) to estimate his volume status. The patient was extubated 48 hours of hospitalization; the DKA was resolved after 52 hours of admission. He was discharged home in good condition on the 5th day. The second patient was a 13-year-old girl, a known case of T1DM, who presented with mixed DKA- hyperosmolar-hyperglycemic state (HHS) and septic shock. She was intubated and treated according to the data derived from pulse Contour Cardiac Output (PiCCO). After 3 days, she was extubated and transferred to the ward in good condition.

**Conclusion:**

Using invasive hemodynamic monitoring in critically ill children with severe DKA and hypotension might guide the physicians for hydration and selecting the most appropriate inotrope.

## Background

Diabetic ketoacidosis (DKA) is one of the hyperglycemic emergencies that may occur in diabetic patients. The diagnostic criteria of DKA include hyperglycemia (blood glucose > 11 mmol/L [198 mg/dL]), venous pH < 7.3 or serum bicarbonate level < 15 mmol/L, and ketonemia (blood β-hydroxybutyrate ≥3 mmol/L) or moderate to severe ketonuria [1]. Another serious complication in diabetic patients is extreme hyperglycemia (blood glucose > 33.3 mmol/L [600 mg/dL]), effective serum osmolality > 320 mmol/kg (mOsmol/kg H2O), venous pH > 7.25, and absent or minimal ketosis, which is defined as hyperglycemic-hyperosmolar state (HHS) [1].

Risk factors of DKA include the lack of taking insulin at the right time or omitting it, history of previous episodes of DKA, inability to drink, having diarrhea, lack of medical follow-up, and infections [2, 3].

The goals of treatment include fluid and electrolyte replacement, as well as correction of acidosis and hyperglycemia. Frequent monitoring of complications such as cerebral edema (CE), venous thrombosis, and acute renal failure, is of paramount importance.

Despite the intracellular and extracellular fluid depletion in DKA, patients have normal or even high blood pressure (BP). Maintaining normal BP is attributed to increased plasma catecholamine concentrations, antidiuretic hormone release, and increased osmotic pressure due to marked hyperglycemia [4]. Measuring the extent of dehydration, which is commonly estimated by physical signs, depends on the examiner’s skill. Therefore, it is not always precise [5]. In children, the mortality rate of DKA is 0.15 to 0.30% in developed countries and up to 13.4% in developing countries [6–8].

DKA with hypotension is uncommon. Therefore, presence of hypotension in a patient with DKA would be a clue for fluid deficit or septic shock. Choosing the correct line of therapy (hydration or inotrope) as well as the right timing would be of great importance in the management of a hypotensive DKA patient.

Invasive hemodynamic monitoring is one of the best means that provides physicians with some parameters to guide the therapy. ProAQT and pulse Contour Cardiac Output (PiCCO) are two invasive continuous hemodynamic monitoring devices used in this regard [9]. These devices provide information to show blood flow, volume responsiveness, afterload, and contractility, as guides to choose the best treatment. PiCCO also gives additional information to estimate volume responsiveness, and parameters indicating pulmonary edema [10].

Herein, we report on two pediatric patients presenting with DKA and septic shock (the second patient was a case with mixed DKA-HHS) monitored by two continuous invasive hemodynamic devices (proAQT and PiCCO). To the best of our knowledge, this is the first effort of using these procedures in pediatric patients with severe DKA and septic shock to guide the therapy according to the hemodynamic parameters.

## Case presentation

### Case I

An 8-year-old boy (weight 21 kg, height 127 cm) was brought to the hospital because of dyspnea and lethargy. He had no previous illness, and was well up to 3 weeks before admission when he had developed polyuria, polydipsia, and increased appetite.

Upon arrival of the patient to the emergency room, his blood sugar was checked to be 21.6 mmol/L (388 mg/dL) (Table 1). His venous blood gas (VBG) showed a pH of 6.8, PCO2 of 22 mmHg, bicarbonate of 3 mmol/L, and base excess (BE) of-31 mmol/L. Urinalysis revealed 3+ ketonuria. On arrival, he had a BP of 100/55 mmHg, heart rate of 133 beats/min, respiratory rate of 47 breaths/min, and oral temperature of 38.8 °C. With the impression of DKA, the patient was transferred to the Pediatrics Ward, and the treatment was started (our treatment is according to International Society for Pediatric and Adolescent Diabetes (ISPAD) Consensus guideline) [1]; so she was hydrated with 10 cc per kilogram normal saline over an hour,and then 10% deficit was planned to be corrected over 48 hour,and intravenous infusion of regular insulin with 0.1 unit per KG. After 5 hours, his VBG revealed a pH of 6.85, PCO2 of 25 mmHg, bicarbonate of 4.6 mmol/L, and BE of − 29.5 mmol/L. His BP decreased to 68/33 mmHg and his heart rate increased to 158 beats/min. Therefore, he was transferred to the Pediatric Intensive Care Unit (PICU).

**Table 1:** The initial laboratory data

Variable along with the reference range	Patient I	Patient II
BUN (mg/dL, 8-20)	15	60
Cr (mg/dL, M: 0.8–1.3, F: 0. 6-1.2)	0.7	1.62
Blood sugar (mg/dL)	388	685
Cl (mEq/L)	114	125
Na (mEq/L)	142	144
K (mEq/L)	4.1	5.2
Mg (mEq/L)	2.5	1.8
WBC (Neutrophil %)	31,800 (87%)	56,800 (70%)
CRP (mg/L, < 6)	> 150	140
Procalcitonin (ng/mL, ≤0.3)	28	35
Albumin (g/dL)	3.3	3.5
CPK (U/L, M: < 171, F: < 145)	83	247
Ca (mg/dL)	9.3	10.2
ALT (U/L, M:< 41, F: < 31)	112	48
AST (U/L, M: < 37, F: < 31)	45	58
LDH (U/L, < 480)	429	987

*Abbreviations*: *BUN* Blood urea nitrogen, *Cr* Creatinine, *WBC* White blood cell count (count/mm3), *CRP* C-reactive protein, *CPK* Creatine phosphokinase, *ALT* Alanine aminotransferase, *AST* Aspartate transaminase, *LDH* Lactate dehydrogenase

In PICU,the Pediatric Sequential Organ Failure Assessment (pSOFA) Score was 9,and as he was in a shock and hypoxic (O2 sat 85%), the patient was intubated in the PICU, and attached to a mechanical ventilator. A central venous catheter was inserted. To closely monitor his hemodynamic, an arterial line connected to a proAQT device, was also inserted into his femoral artery. According to the device data (Table 2), the patient was hydrated with 20 mL/kg of normal saline, and broad-spectrum antibiotics were started. Afterward, inotrope (epinephrine) was started, and 1 mEq/kg of sodium bicarbonate was given over 1 hour.

**Table 2:** The initial hemodynamic indices

Variables (Unit, Reference range)	Patient I	Patient II
Heart rate (beats/min)	158	165
Central venous pressure, mmHg	5	4
SCVO2	72%	68%
Systolic BP (mmHg)	62	75
Diastolic BP (mmHg)	35	42
MAP (mmHg)	44	53
CI (L/min/m2)	1.2	5.8
ITBI	N/A	468
ELWI (mL/kg, 3–7)	N/A	7
GEDI (mL/m2, 680–800)	N/A	375
SVRI (1700–2400)	3420	574
PPV (%, 0–10)	17	18
SVV (%, 0–10)	15	22
PVPI (1–3)	N/A	2.1

*Abbreviations*: *SCVO2* Central venous O2 saturation, *MAP* Mean arterial blood pressure, *CI* Cardiac index, *ITBI* Intra-thoracic blood volume index, *EVLWI* Extravascular lung water index, *GEDI* Global end-diastolic index, *SVRI* Systemic vascular resistance index, *PPV* Pulse pressure variation, *SVV* Stroke volume variation, *PVPI* Pulmonary vascular permeability index

On PICU arrival, the parameters derived from the invasive monitoring device of the first patient were mean arterial pressure (MAP 41 mmHg), systemic vascular resistance index (SVRI 2421), stroke volume variation (SVV 15%), pulse pressure variation (PPV 17%), and cardiac index (CI 1.2 [normal range 2.5–4.0] L/min/m2) (Table 2). The parameters showed that the patient was volume responsive. Therefore, he was hydrated with 20 mL/kg of normal saline over 15 min. In addition to hourly fluid administration, as maintenance and deficit (12%), inotrope (epinephrine) infusion was started regarding the low CI and increased SVRI. Sodium bicarbonate was infused (1 mEq/kg) over 1 h because the pH was < 7.00. After a while, the parameters changed to a MAP of 65 mmHg, SVV of 12%, PPV of 13%, and urine output of 2 mL/kg/hr. At this time, we preferred to follow the patient’s VBG, urine output, and hemodynamic parameters and hydrate the patient only with maintenance and deficit fluid instead of additional bolus hydration because of the increased risk of CE. The patient’s clinical condition (i.e., BP, urine output, VBG, and hemodynamic data) was improved. Thirty hours after the treatment, epinephrine infusion was decreased to 0.05 μg/kg/min. Finally, the hemodynamic parameters were SVV of 8%, PPV of 9%, MAP of 64 mmHg, and CI of 3.5 L/min/m2. The inotrope was tapered at the 36th hour and then discontinued.

The patient’s blood pressure and venous blood gas improved (pSOFA score was 3 in the 2nd day). He was extubated after 48 hrs. About 4 h later, pH was more than 7.35; serum bicarbonate, > 15 mmol/L. Finally, on the 4th day, the patient was discharged from PICU in good condition without any sequelae.

### Case II

A 13-year-old girl (weight 45 kg, height 155 cm) who was a known case of T1DM for 5 years, was brought to the hospital due to decreased level of consciousness. She was feverish and lethargic in the previous 2 days, and thus she had not used insulin.

In the emergency room, she had a BP of 82/35 mmHg, heart rate of 168 beats/min, respiratory rate of 48 breaths/min, and oral temperature of 39.5 °C. Her Glasgow coma scale score was six. The blood sugar was checked with a glucometer, which was high(plasma glucose level was 685 mg/dL). Urine ketone was 2+. VBG revealed a pH of 6.67, PCO2 of 15 mmHg, bicarbonate of 1 mmol/L, and BE of-32 mmol/L (Table 1).

The patient was hydrated with 20 mL/kg of normal saline, and intubated; sodium bicarbonate 1 mEq/kg was also given twice, and empiric antibiotic started within a first hour. Then, she was transferred to PICU with pSOFA scoe 9. In the PICU, norepinephrine was started, sodium bicarbonate was given due to acidemia (pH 6.82), and again she was hydrated with 20 mL/kg of normal saline, and infusion of regular insulin (0.1 unit per KG) was started after an hour. According to the laboratory data (Table 1), the patient had mixed DKA-HHS. Therefore, a deficit of 12% was added to the maintenance fluid. Based on the data obtained from the device, vasopressin was added to norepinephrine, and the patient was hydrated three times over the next 24 hours.

To better monitoring of her hemodynamic, a thermodilution arterial line of PiCCO was inserted into her femoral artery. In addition, a central venous catheter was inserted into the internal jugular vein connected to the monitor. It was calibrated with cold saline (Table 2).

The patient had elevated CI and decreased SVRI (Table 2). In addition to hydration and administration of sodium bicarbonate, norepinephrine administration was started due to decreased SVRI. Vasopressin was also added, although it is not commonly used in pediatric patients. The degree of deficit was increased to 18%. The inotropes were tapered, and the pH was increased. Eventually, her condition improved so that in the 42nd hour of admission, her VBG revealed a pH of 7.38 and a bicarbonate of 15.2 mmol/L(pSOFA score was 4). On the 3rd day, she was extubated, and on the 5th day, she was transferred to the Pediatrics Ward in good condition.

## Discussion and conclusions

We presented two patients with DKA and septic shock. They both had severe DKA, hypotension, and hypoxemia. Their hemodynamic variables were measured using invasive hemodynamic monitoring devices. The recommendation of the International Surviving Sepsis Campaign for children is to employ advanced hemodynamic indices, besides clinical data [11]. Although there are several methods of invasive hemodynamic monitoring in our center, we used two less invasive methods—ProAQT and PiCCO. PiCCO provides more hemodynamic parameters to assess volume status and variables to determine the volume overload [10].

Regarding the issue of volume depletion in DKA, the main goal of fluid therapy is preserving adequate circulation to the vital organs and restoring the deficiency of the fluid over 24–48 hrs. Both overhydration and underhydration are harmful, management of patients with DKA and hypotension would be more complicated. Invasive hemodynamic monitoring should thus be used to determine when to start hydration and how much fluid should be given to the patient, when to stop hydration, and which inotrope should be initiated [11, 12].

Leukocytosis is generally seen in patients with DKA. However, when white blood cell counts are > 20,000/μL, we should consider sepsis and start appropriate antibiotics as delayed treatment increases the mortality [13, 14]. Our patients had leukocytosis of more than 20,000/μL; they were clinically suspected to have infection. The causative organisms in blood cultures were Streptococcus and Klebsiella spp. in the first and second patients, respectively; antibiotic was started in the first hour of admission in the second patient, but it was started on the 6th hour of admission in the first case when he was admitted in PICU.

Hypovolemia and sepsis are the main factors in patients with DKA and shock. However, it is not easy to differentiate these two solely based on physical examinations. It often seems better to use a combination of these two, but it is essential to determine what to choose for increasing the BP (fluid vs inotrope) or which inotrope should be started. Although hydration of the patient with more than 30 mL/kg in the first 8 h is rarely recommended in DKA management [1, 11], our first patient received 50 mL/kg of hydration in the first 2 h; the second received up to 60 mL/kg.

There is a controversy about the etiology of CE. Some suppose that rapid fluid administration with rapid changes in serum osmolality is responsible for this disorder [15]. Therefore, CE was considered during the treatment and after the first hydration, as the patient’s hemodynamic parameters improved. According to SVV, we could give him bolus hydration, but we did not. The reason was that we preferred to use laboratory data along with these hemodynamic variables.

The higher mortality rate in patients with DKA in underdeveloped countries can be attributed to sepsis, septic shock, delayed recognition, CE, and renal failure [16].

For managing patients with severe DKA and hypotension, we cannot solely rely on clinical examination and laboratory data to save the vital organs. Underhydration may result in organ failure such as renal failure; overhydration, on the other hand, may lead to pulmonary and Especially in other situations such as: DKA with renal failure or heart failure which we can not hydrate the patients as routine, Therefore, it would be better to use invasive hemodynamic monitoring or even non- invasive tools (according to availability of equipment in each center) in critically ill patients to guide the physicians for hydration and selecting the most appropriate inotrope;but To date, there are no experiments that investigated the acceptable range of hemodynamic parameters to guide fluid therapy in patients with DKA,or DKA with shock; so additional studies are required to identify the safe ranges.

## Data Availability

Data will be shared on the reasonable request to the corresponding author.

## Abbreviations

AST: Aspartate transaminase
ALT: Alanine aminotransferase
BP: Blood pressure
BUN: Blood urea nitrogen
CE: Cerebral edema
CI: Cardiac index
CPK: Creatine phosphokinase
Cr: Creatinine
CRP: C-reactive protein
DKA: Diabetic ketoacidosis
EVLWI: Extravascular lung water index
GEDI: Global end-diastolic index
ITBI: Intra-thoracic blood volume index
LDH: Lactate dehydrogenase
MAP: Mean arterial blood pressure
PiCCO: Pulse contour cardiac output
PICU: Pediatric intensive care unit
PPV: Pulse pressure variation
PVPI: Pulmonary vascular permeability index
SCVO2: Central venous O2 saturation
SVRI: Systemic vascular resistance index
SVV: Stroke volume variation
T1DM: Type 1 diabetes mellitus
VBG: Venous blood gas
WBC: White blood cell count
